# Rectal Foreign Body Due to Accidental Ingestion of Denture Fragments in an Elderly Patient

**DOI:** 10.7759/cureus.90252

**Published:** 2025-08-16

**Authors:** Kenta Mitsusada, Hisashi Dote, Yuichiro Miyaki, Yosuke Yamada

**Affiliations:** 1 Department of Surgery, Seirei Hamamatsu General Hospital, Hamamatsu, JPN; 2 Department of Emergency and Critical Care Medicine, Seirei Hamamatsu General Hospital, Hamamatsu, JPN; 3 Department of Gastroenterology, Seirei Hamamatsu General Hospital, Hamamatsu, JPN

**Keywords:** accidental ingestion, cognitive impairment and dementia, denture, endoscopy, gastrointestinal foreign body

## Abstract

Foreign body ingestion is an important yet often overlooked cause of gastrointestinal symptoms in elderly patients, particularly those wearing dentures. Atypical presentations due to reduced visceral pain sensitivity and cognitive impairment may delay recognition and increase complication risk.

A man in his late 80s presented with two weeks of lower abdominal pain. He had diabetes mellitus, angina pectoris, chronic obstructive pulmonary disease, and hypothyroidism, and no history of gastrointestinal surgery or prior swallowing incidents. Physical examination showed mild lower abdominal tenderness without peritoneal signs. CT demonstrated a high-attenuation linear structure in the rectosigmoid region with surrounding fat stranding. Given his comorbidities and surgical risk, colonoscopic retrieval was performed, and an approximately 5 cm denture fragment was removed without perforation. He recovered uneventfully and was discharged on day 6.

Foreign body ingestion in elderly patients often involves dental prostheses. Prior reports frequently required surgery due to perforation, whereas early CT diagnosis and careful endoscopic removal can enable minimally invasive management, even in rectosigmoid cases. Although endoscopic success rates are high in aggregate, lower gastrointestinal cases remain rare.

In elderly denture wearers with unexplained abdominal pain, accidental ingestion should be considered early. CT imaging and denture inspection can facilitate prompt diagnosis. When feasible, minimally invasive endoscopic retrieval may avoid surgery and achieve favorable outcomes in selected high-risk patients.

## Introduction

Diagnosing abdominal disease in elderly patients is challenging due to the frequent absence of characteristic symptoms or the presence of nonspecific presentations [1]. Cognitive impairment and communication barriers can further reduce the reliability of clinical interviews and obscure key findings, leading to delayed or missed diagnoses [2,3]. Accidental ingestion of foreign bodies, including denture fragments, is a clinically significant but often underrecognized cause of abdominal symptoms in this population and can lead to severe complications if not promptly identified [4-6].

## Case presentation

A man in his late 80s was referred for evaluation of persistent lower abdominal pain of two weeks’ duration. Past medical history included diabetes mellitus, angina pectoris, chronic obstructive pulmonary disease, and hypothyroidism. Regular medications were metformin, teneligliptin, aspirin, cilostazol, tiotropium bromide, and levothyroxine. He had no history of gastrointestinal surgery and no prior swallowing incidents. On presentation, he was afebrile with stable vital signs. Physical examination revealed mild lower abdominal tenderness without peritoneal signs. Initial laboratory results were as follows: white blood cell 6,750/μL, C-reactive protein 0.73 mg/dL, sodium 131 mEq/L, potassium 3.6 mEq/L, chloride 98 mEq/L, blood urea nitrogen 16 mg/dL, and creatinine 1.2 mg/dL.

**Figure 1:**
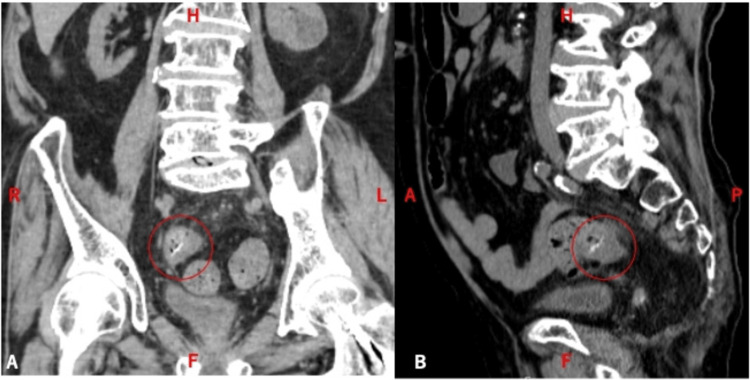
(A) Coronal and (B) sagittal CT showing a linear high-attenuation structure in the rectosigmoid colon (circle) CT: computed tomography

Non-contrast abdominal CT showed a high-attenuation linear structure in the rectosigmoid region with surrounding fat stranding (Figure 1).

**Figure 2:**
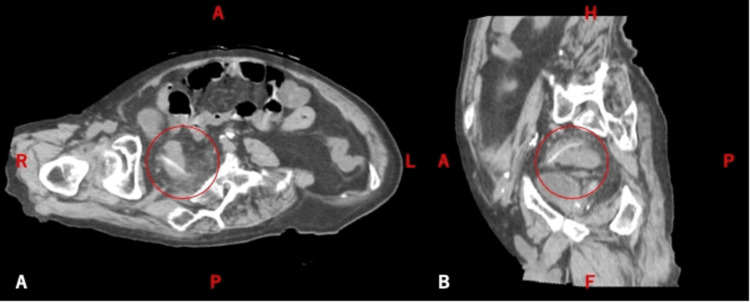
CT reconstruction showing surrounding fat stranding; the tip of the foreign body appears to extend beyond the bowel wall (circle) CT: computed tomography

Three-dimensional reconstruction suggested that the tip may have protruded beyond the intestinal wall, raising concern for microperforation (Figure 2).

**Figure 3:**
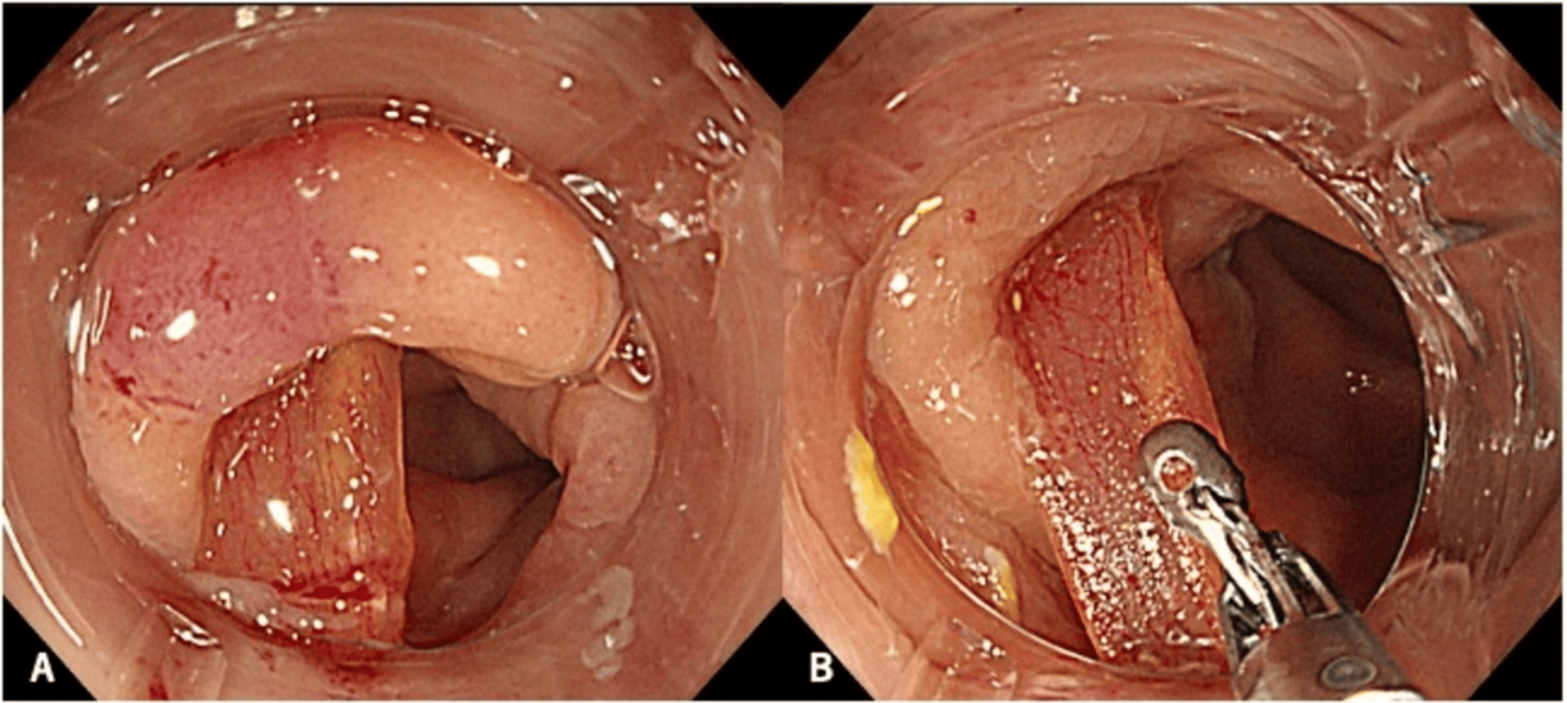
(A, B) Endoscopy showing mucosal edema and erythema in the rectosigmoid region and removal with forceps

Based on the imaging findings and the patient’s comorbidities, which conferred elevated operative risk and potential need for permanent colostomy, minimally invasive endoscopic retrieval was prioritized. Colonoscopy demonstrated mucosal edema and erythema from the sigmoid colon to the rectosigmoid junction, with a linear foreign body embedded in the mucosa (Figure 3).

**Figure 4:**
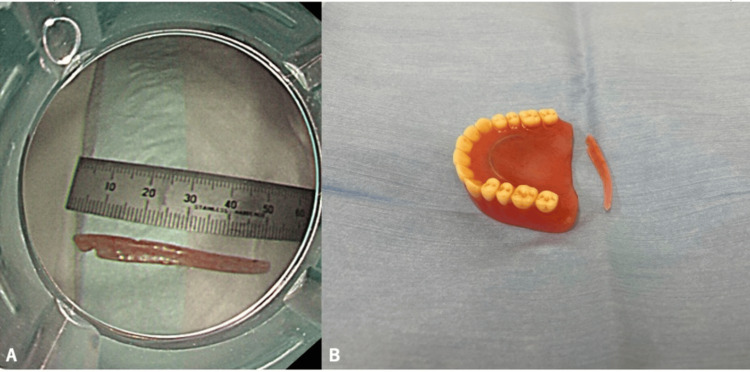
(A, B) Extracted denture fragment, approximately 5 cm in length

Using grasping forceps under careful visualization, we removed an approximately 5 cm denture fragment intact (Figure 4).

A repeat CT after the procedure showed no free air or new findings. We administered tazobactam/piperacillin for four days due to concern for microperforation, resumed diet on hospital day 3, and discharged the patient on day 6 without complications.

## Discussion

Epidemiology and diagnostic challenge

Elderly individuals often present atypically due to reduced visceral pain sensitivity and cognitive dysfunction, masking the severity of underlying gastrointestinal disease and limiting the reliability of ingestion history [1-3]. More than 70% of ingested foreign bodies in elderly patients involve dental prostheses, underscoring denture use as a significant risk factor in this population [7].

Imaging and early recognition

Plain radiographs may fail to detect denture fragments due to radiolucent components; therefore, CT is indispensable for detection, localization, and complication assessment (e.g., fat stranding, microperforation) [8,9]. In our case, the distinct high-attenuation linear structure with surrounding fat stranding on CT was supported by 3D reconstruction-guided prompt endoscopic management.

Management strategy and comparison with the literature

Prior reports have described denture ingestion leading to small or large bowel perforation, with most cases requiring laparotomy or laparoscopy and, at times, stoma formation [8,9]. In contrast, our case demonstrates that early CT-based recognition and careful colonoscopic retrieval can avoid surgery even for rectosigmoid impaction. Although a retrospective series reported high endoscopic success rates (>90% for upper endoscopy and 100% for colonoscopy), reports specifically describing lower gastrointestinal denture retrievals remain limited [10]. Our case thus adds to the small body of evidence supporting minimally invasive management in carefully selected elderly patients at high operative risk.

Clinical take-home points

In elderly denture wearers with unexplained abdominal pain, ingestion should be ruled out early. CT is valuable for both diagnosis and assessment of complications. When feasible and safe, endoscopic retrieval should be considered as the first-line approach, even for lower gastrointestinal foreign bodies, as it may avoid surgery and help preserve quality of life.

Limitations

This is a single case report; therefore, its generalizability is limited.

## Conclusions

In elderly patients wearing dentures who present with unexplained abdominal pain, accidental ingestion of denture fragments should be considered. Early CT imaging and denture inspection can aid prompt diagnosis, allowing minimally invasive treatment in selected cases and potentially avoiding surgery. This case illustrates that even rectosigmoid denture impaction can be managed endoscopically with favorable outcomes in high-risk elderly patients.
